# Physical activity and sedentary pattern compositions and cardiometabolic risk in preschoolers

**DOI:** 10.1186/s44167-025-00090-y

**Published:** 2025-12-01

**Authors:** Katherine L. Downing, Simone J. J. M. Verswijveren, Lisa Bell, Peter Vuillermin, David Burgner, Anne-Louise Ponsonby, Martin O’Hely, Anna Timperio, Jo Salmon, Kylie D. Hesketh

**Affiliations:** 1 Institute for Physical Activity and Nutrition (IPAN), Deakin University, Geelong, VIC Locked Bag 20001, 3220 Australia; 2 Royal Children’s Hospital, Murdoch Children’s Research Institute, University of Melbourne, Melbourne, VIC Australia; 3 Child Health Research Unit, University Hospital, Barwon Health, Geelong, VIC Australia; 4 School of Medicine, Deakin University, Geelong, VIC Australia; 5 The Florey Institute of Neuroscience and Mental Health, University of Melbourne, Melbourne, VIC Australia

**Keywords:** Physical activity, Sedentary time, Cardiometabolic risk, Preschool children, Bouts, Compositional analysis, Time-use patterns

## Abstract

**Background:**

There is increasing interest in the importance of patterns of accumulation and overall daily time-use composition of physical activity (PA) and sedentary time (SED) for children’s cardiometabolic health. This study examined cross-sectional associations between the time-use composition of PA and SED patterns with cardiometabolic risk factors in 4-year-olds.

**Methods:**

Data were drawn from the Barwon Infant Study 4-year review (*n* = 467). Accelerometer data were classified into short (≤ 1-minute) and long (> 1-min) SED, light-, moderate-, and vigorous-intensity PA (LPA, MPA, VPA) bouts. A waking time-use composition of eight distinct components (total volumes plus short and long bouts of SED, LPA MPA, VPA) was constructed using compositional data analysis. Linear mixed models examined associations between composition patterns and body mass index (BMI), percent body fat, triceps and subscapular skinfold thickness, blood pressure, heart rate, carotid-femoral pulse wave velocity, and aortic and carotid intima-media thickness.

**Results:**

Adjusted models indicated a higher ratio of long versus short LPA bouts was associated with higher z-BMI (β = 1.69, SE = 0.83, *p* = 0.04), percent body fat (β = 10.72, SE = 3.71, *p* = 0.004), and z-triceps (β = 1.90, SE = 0.93, *p* = 0.04). A higher ratio of long versus short MPA bouts was associated with lower z-BMI (β = − 0.99, SE = 0.46, *p* = 0.03) and percent body fat (β = − 4.63, SE = 1.93, *p* = 0.02). A higher total volume of MPA versus VPA was associated with higher percent body fat (β = 4.07, SE = 1.63, *p* = 0.01) and z-triceps (β = 1.05, SE = 0.43, *p* = 0.01). Other outcomes showed no associations (*p* ≥   0.05).

**Conclusions:**

In preschoolers, accumulating LPA in shorter bursts, MPA in longer bursts, and maintaining a higher proportion of VPA may support healthier adiposity profiles. These findings underscore the importance of minimizing prolonged sedentary time and encouraging sustained, high-intensity PA from early childhood.

**Supplementary Information:**

The online version contains supplementary material available at 10.1186/s44167-025-00090-y.

## Background

It is well established that participating in recommended amounts of physical activity (PA) and limiting sedentary time (SED; e.g., prolonged sitting or screen viewing) during early childhood are important for health and development [1, 2]. There is also emerging evidence on the importance of patterns of accumulation (i.e., ‘bouts’) of PA and SED for children’s cardiometabolic health [3]. In adults, bouts of moderate- to vigorous-intensity PA (MVPA) for more than 10 min are associated with reduced risk of mortality [4]. Conversely, longer bouts of SED are associated with increased risk of mortality,^5^ and interspersing them with light-intensity PA (LPA) is associated with lower adiposity and postprandial glycaemia [6]. However, there are limited data examining the influence of patterns of accumulation on cardiometabolic health in early childhood (birth through 5 years). Johannsson et al. (2015) found no differences in the frequency or total time spent in bouts of SED, ‘low-intensity PA’ and ‘high-intensity PA’ in a small sample of normal weight (*n* = 104) and overweight (*n* = 16) toddlers (aged 2 years) [7]. Similarly, Viegas et al. (2023) [8] reported no differences in the frequency or average time spent in bouts of MVPA between healthy weight (*n* = 25) and overweight (*n* = 25) preschoolers (aged 3–5 years). However, in a sample of toddlers and preschoolers (*n* = 100; mean age 3 years), Kuzik and Carson (2016) [9] found that time spent in 1–4-minute SED bouts was significantly associated with BMI z-scores in unadjusted analyses, but borderline non-significant (*p* = 0.05) after adjusting for child age, sex and parental education.

Moreover, SED and PA provide a partition of an individual’s waking day. When time in one movement behavior increases, less time may be available for another [6]. The field has primarily investigated associations between separate movement behaviors of different intensities and health, but there is mounting evidence that the overall daily time-use composition of behaviors may be more important for health [6] and more comprehensively reflects how activity is accumulated. In this context, compositional data analysis can be used to examine the relative distribution of time spent in SED, LPA, moderate-intensity PA (MPA) and vigorous-intensity PA (VPA) across a 24-hour or waking day. A 2025 systematic review and meta-analysis [10] examining associations between 24-hour movement behaviours and cardiometabolic risk factors across early childhood to adolescence identified 12 relevant studies that included preschool-aged or younger children (out of 38 total). Of these, nine studies investigated associations between meeting the guidelines and indicators of body size and composition, with mixed findings: most reported either null associations or associations indicating lower body mass index (BMI) among children meeting the guidelines. Only one study included cardiometabolic outcomes beyond body composition, specifically high-density lipoprotein cholesterol (HDL-C), triglycerides, glucose, and systolic blood pressure [11]. In that study, Vanderloo et al. [11] found that meeting all movement guidelines was associated only with HDL-C. The remaining three studies included in the review employed either compositional data analysis [12, 13] or latent profile analysis [14] to explore associations between movement behaviours and cardiometabolic health. All three focused exclusively on body size and composition measures and reported associations between behavioural composition and BMI. These findings highlight that, while some evidence exists regarding associations between movement behaviors and body composition in preschool-aged children, both the use of compositional data analyses and the investigation of cardiometabolic outcomes beyond adiposity remain relatively limited in this young population.

A study in older children employed compositional data analysis to examine how different patterns of physical activity accumulation were associated with cardiometabolic health, finding that accumulating PA (particularly LPA and VPA) in frequent short bursts may be more beneficial for adiposity markers than sporadic continuous activity [15]. This highlights the need to consider both the composition of movement behaviors and their accumulation patterns when investigating cardiometabolic risk. Therefore, the aim of this study was to examine cross-sectional associations between daily waking time-use compositions of PA and SED patterns and cardiometabolic risk factors in 4-year-old children.

## Methods

The Barwon Infant Study (BIS) is a birth cohort study (*n* = 1074) recruited using an unselected sampling frame in south-eastern Australia [16]. The current study utilized data from the preschool (4-year) review, conducted from February 2014 to February 2017. The study was approved by the Barwon Health and Deakin University Human Research Ethics Committees (Barwon Health HREC approved item number: 10.24). Parents provided written, informed consent prior to data collection.

### Accelerometry

Children were fitted with GT3X ActiGraph accelerometers (Pensacola, FL, USA) on an elastic belt at the right iliac crest. They were instructed to wear them during waking hours, removing them for sleep and water-based activities (e.g., bathing, swimming), for seven consecutive days. Data were collected in 15-second epochs and non-wear time was determined as at least 20 min of consecutive zero counts [17]. Inclusion criteria were at least six hours of accelerometer wear per day on at least four days [18]. Epochs were classified as SED if they had fewer than 26 counts [19], LPA if they had 26–419 counts, MPA if they had 420–841 counts, and VPA if they had 842 or more counts [20]. Total volumes of SED, LPA, MPA and VPA were then collapsed into time spent in ‘short’ and ‘long’ bouts. First, the median bout length above 0.25 min (i.e., one 15-second epoch) was determined, to give some indication of the favored length [21] for each of the intensities. As all observed sample median bout lengths were below 1 min (approximately 0.25 for MPA and VPA, 0.45 for LPA, and 0.50 for SED), long bouts were defined as more than 1 min for each intensity. Time in short bouts was calculated by subtracting time in long bouts from the total time in the respective intensity. No interruptions in intensity were allowed.

### Cardiometabolic risk factors

Trained researchers collected anthropometric and cardiometabolic measures, including height, weight, percent body fat, triceps and subscapular skinfolds, blood pressure, heart rate, pulse wave velocity (PWV), and aortic and carotid intima-media thickness (aIMT and cIMT, respectively), during the single, on-site preschool review attended by the children and their parents. Height and weight measures were taken in light clothing without shoes or socks. For all measures of adiposity (except percent body fat), two measurements were taken and a third was recorded if the results differed by more than 10%. The average of the two closest measurements were used in analyses. Height (m) was measured using a stadiometer (Seca 206) and weight (kg) using top loading scales (Tanita BC 420 MA). Body mass index (BMI) was calculated (kg/m^2^) and age- and sex-standardized z-scores (zBMI) were determined according to the World Health Organization (WHO) Reference 2006 charts [22]. Percent body fat was measured using Bioelectric Impedance Analysis body composition monitors (Tanita BC 420 MA). Triceps and subscapular skinfold thickness (mm) were measured using Holtain calipers and converted to age- and sex-standardized z-scores according to the WHO Reference 2007 charts [23]. Brachial blood pressure (mmHg), heart rate (beats per minute; BPM), and carotid-femoral PWV (meters per second; m/s) were averaged across three readings in a resting, supine position using SphygmoCor XCEL (AtCor Medical), and mean far wall aIMT and maximum far wall cIMT (mm) were quantified following ultrasound imaging using the GE Vivid-I (GE Healthcare), as previously described [24, 25]. 

### Covariates

Date of birth, sex, and birth weight were obtained from hospital records. Birth weight z-scores standardized for sex and gestational age were calculated according to the WHO Reference 2006 charts [22]. Mothers reported their highest level of education at baseline, categorized as: year 10 or equivalent; completion of high school, trade, certificate, apprenticeship, or diploma; and tertiary education. At the preschool review, parents reported their child’s usual dietary intake using a food frequency questionnaire [26], from which total energy intake (kJ) was calculated.

### Statistical analyses

Analyses were undertaken in Stata 15.0 (StataCorp, College Station, TX, USA) and RStudio 3.5.1 (RStudio Inc., Boston MA), using the packages ‘compositions’ (‘acomp’ framework) and ‘stats’ (‘lm’ function) [27, 28]. The proportions of the 24-hour day spent in different intensities were normalized for each participant such that their sum equaled one. A time-use composition of eight distinct components (time in total volumes and shorter and longer bouts of SED, LPA MPA and VPA) was constructed using compositional data analysis. The accelerometry data contained no zeros, so zero replacement was not required. Standard descriptive statistics and the means of percentages of total wear time of each of the components were reported for participants with complete, valid accelerometry data.

**Fig. 1:**
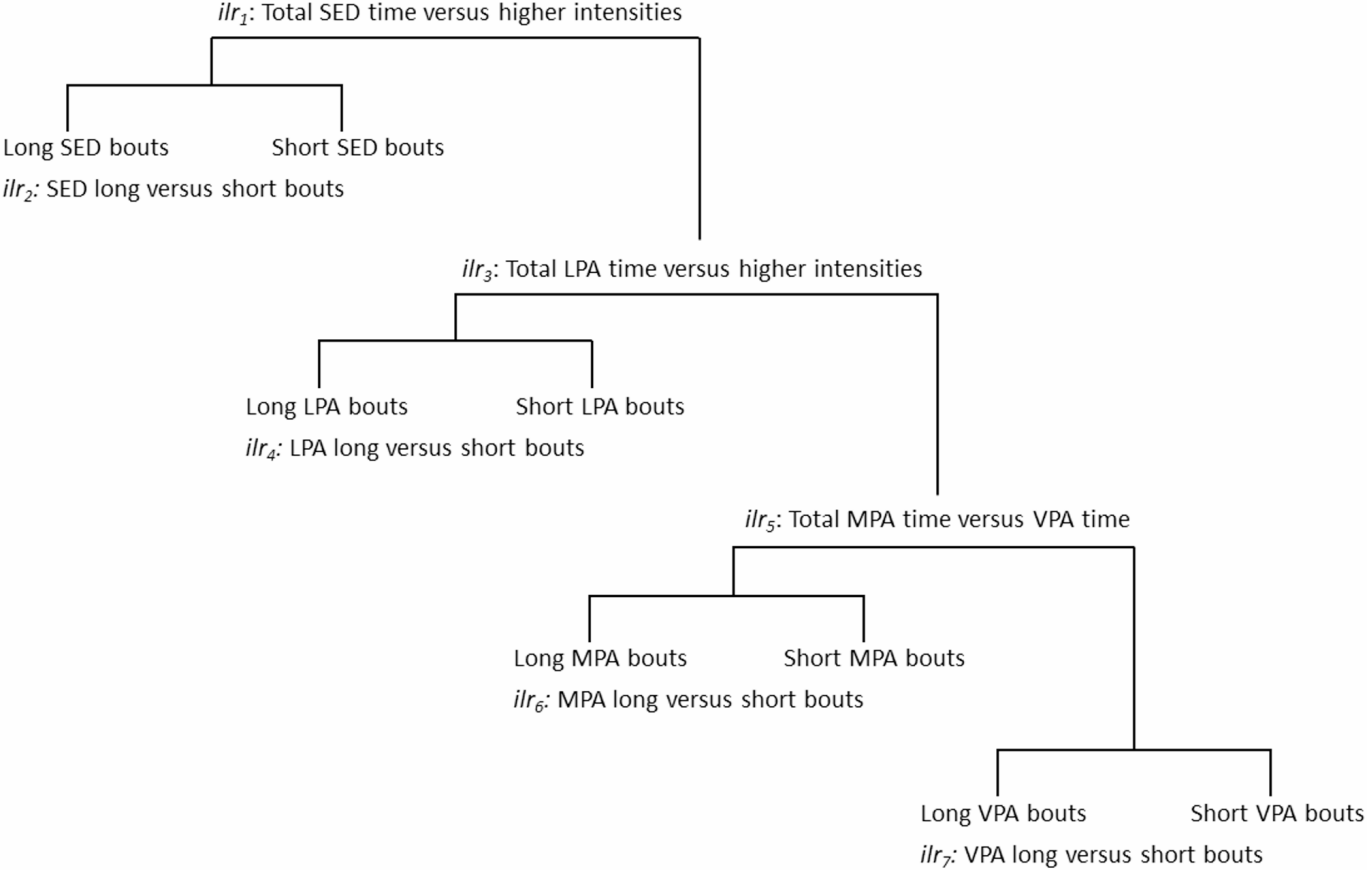
Visual representation of the sequential binary partition. *Abbreviations* *ilr *, isometric log-ratio; LPA,  light-intensity physical activity; MPA, moderate-intensity physical activity; SED, sedentary time; VPA, vigorous-intensity physical activity

The eight-part time-use composition was modelled as a set of seven isometric log-ratio (*ilr*) coordinates [29–31]. Specifically, the seven *ilr* coordinates were set up using a sequential binary partition (see Fig. 1 and Supplementary Table 1) that allowed investigation of relative volumes of each intensity versus time in the remaining intensities (e.g., *ilr* coordinate 1 represents the SED time versus time in remaining intensities), as well as direct comparisons of time spent in longer versus shorter bouts in each specific intensity (e.g., *ilr* coordinate 2 represents the longer versus shorter sedentary bouts) [15]. This ensures that regression estimates of each bout length (longer versus shorter) are controlled for both other bout durations and the relative volumes of each intensity.

Linear regression models were used to test associations between the time-use composition (including all seven *ilr* coordinates simultaneously as the exposure) and the cardiometabolic risk factors as outcomes, adjusting for sex (categorical), age at physical measurement, maternal education (categorical), total energy intake, and birth weight z-score. The F-statistic and corresponding p-value gives an indication of the overall association between the time-use composition and the outcomes (BMI z-score, percent body fat, triceps skinfold z-score, subscapular skinfold z-score, pulse wave velocity, systolic blood pressure, diastolic blood pressure, heart rate, aIMT and cIMT). In addition, individual regression coefficients for each of the seven *ilr* coordinates were reported in the one model. The *ilr* parameters 1, 3 and 5 correspond to relative proportions of SED, LPA, and MPA versus the remaining (higher/more intense) intensities, respectively. In contrast, the *ilr* parameters 2, 4, 6 and 7 correspond to relative proportions of long versus shorter bouts of SED, LPA, MPA and VPA, respectively. All parameters were obtained within the same time-use composition models. Statistical significance was set at *p* < 0.05. All models were repeated with a subsample excluding participants considered ‘too thin’ based on previously published sex-specific percentile curves for 5-year-olds, as no younger age percentile curves are available (2nd percentile, 13.8% for girls and 12.2% for boys [32]).

To further investigate the observed association between the compositions and percent body fat, the significant compositional model was used to estimate the ‘Goldilocks Day’ time-use composition [33] associated with the highest 5% and lowest 5% percent body fat measurements (i.e., the 23 ‘worst’ and ‘best’ measuring participants, respectively). To apply the Goldilocks Day method, we used our fitted compositional regression model to predict body fat percentage (BF%) for each observed minute-level composition in our dataset, holding covariates at their sample means and giving equal weight to boys versus girls. From these model-predicted values, we identified the lowest 5% (“best”) and highest 5% (“worst”) of predicted BF% across the *observed* compositions. We then calculated the compositional (closed) mean of each of these two subsets and rescaled it to a waking-day total of 660 min to express the results in minutes per behaviour. This was based on the sample average wear time (662 min) and in line with sleep recommendations of uninterrupted 10–13 h of sleep every day for those aged 3–5 years [34]. The Goldilocks Day compositions represent the observed patterns associated with the most and least favourable predicted body fat profiles. Participants (*n* = 10) who were considered ‘too thin’ based on sex-specific percentile curves [32] were excluded from the Goldilocks Day analysis, given that may not be considered to be in optimal health in terms of their percent body fat.

## Results

Of the 650 children who returned the accelerometer, 482 children met the ≥ 6 h of wear time on ≥ 4 days criterion; these data were used to form daily time-use compositions. The included children were on average 4.2 years of age and 65% of mothers had a tertiary education (Table 1). Outcome data were available for between 315 and 475 children with valid accelerometry and outcome data; we conducted separate complete case analyses for each outcome of interest to maximize the sample sizes. Standard descriptive statistics and compositional means of the proportion of time spent in short and long bouts of SED, LPA, MPA and VPA are presented in Table 2. The median (IQR) accelerometer wear time was 660 (89) min/day. Children spent on average approximately 48% of their wear-time in SED, and 38%, 10%, and 4% in LPA, MPA and VPA, respectively. Approximately 75% of children’s total SED time was spent in long bouts > 1 min. In comparison, the respective proportions of time spent in long LPA, MPA and VPA bouts were 48%, 12%, and 26% respectively.

**Table 1:** Participant characteristics

	*n*	Summary statistic
*Child characteristics*		
Sex (female), n (%)	479	228 (48)
Age (years), mean ± SD	482	4.2 ± 0.2
Maternal education, n (%)	481	
Year 10 or equivalent		20 (4)
Year 12, trade, certificate, apprenticeship, or diploma		158 (33)
Tertiary		303 (63)
Total energy intake (kJ), mean ± SD	476	5721.3 ± 1986.9
Birth weight z-score, mean ± SD	478	0.5 ± 0.9
*Outcomes*		
BMI z-score, mean ± SD	475	0.6 ± 0.9
Percent body fat, mean ± SD	408	19.6 ± 3.2
Triceps skinfold z-score, mean ± SD	434	0.4 ± 0.9
Subscapular skinfold z-score, mean ± SD	423	− 0.08 ± 1.1
Systolic blood pressure (mmHg), mean ± SD	315	106.8 ± 8.4
Diastolic blood pressure (mmHg), mean ± SD	315	64.7 ± 6.9
Heart rate (BPM), mean ± SD	314	89.0 ± 9.5
Pulse wave velocity (m/s), mean ± SD	347	4.0 ± 0.5
Mean far aortic intima-media thickness (mm), mean ± SD	318	0.5 ± 0.006
Maximum far carotid intima-media thickness (mm), mean ± SD	379	0.5 ± 0.005

BPM, beats per minute; m/s, metres per second; mm, millimetre; mmHg, millimetre of mercury; kJ, kilojoules; SD, standard deviation

**Table 2:** Standard descriptive statistics and compositional means of the proportion of time spent in short and long bouts of SED, LPA, MPA, and VPA (*n* = 482)

	Standard descriptive statistics, min/day				Compositional mean, % of wear time*
	Q1	Median	Q3	Range	Mean
*SED*					
Short	71.0	78.8	86.7	42.6–115.4	12.0
Long	201.9	228.8	268.3	105.3–599.8	36.0
*LPA*					
Short	121.7	132.3	144.9	82.7–186.2	20.1
Long	103.0	120.0	136.2	51.5–202.3	18.2
*MPA*					
Short	47.2	56.2	66.6	20.8–107.8	8.6
Long	5.5	7.5	10.3	1.5–27.1	1.2
*VPA*					
Short	13.3	18.1	23.7	4.8–56.1	2.9
Long	3.0	5.2	8.8	0.1–33.8	0.9

* Median (IQR) wear time was 660.3 (89.3) min/day

LPA, light-intensity physical activity; min, minutes; MPA, moderate-intensity physical activity; SED, sedentary time; VPA, vigorous-intensity physical activity

Results from the linear regression analyses modelling the association between the time-use compositions, including the seven *ilr* coordinates, and health outcomes are provided in Table 3. Models excluding the ten participants that were considered ‘too thin’ obtained similar results (results not shown). The overall time-use composition was associated with all adiposity markers (i.e., BMI z-score, percent body fat, triceps skinfold z-score and subscapular skinfold z-score), heart rate, aIMT and cIMT. The individual *ilr* coordinates within these models showed that a higher ratio of long vs. short LPA bouts was associated with higher BMI z-score (β = 1.70, standard error [SE] = 0.83, *p* = 0.04), percent body fat (β = 11.20, SE = 3.70, *p* = 0.003), and triceps z-score (β = 2.06, SE = 0.92, *p* = 0.03). Conversely, a higher ratio of long vs. short MPA bouts was associated with lower BMI z-score (β=-1.03, SE = 0.46, *p* = 0.03), percent body fat (β = − 5.28, SE = 1.90, *p* = 0.006), and triceps z-score (β = − 1.03, SE = 0.50, *p* = 0.04). A longer total volume of MPA vs. total volume of VPA was associated with higher percent body fat (β = 4.57, SE = 1.61, *p* = 0.005) and triceps z-score (β = 1.0, SE = 0.42, *p* = 0.007).

**Table 3:** Associations of time-use compositions with cardiometabolic risk factors in 4-year-old children

		Total SED vs. higher intensities [ilr1]		SED long vs. short bouts [ilr2]		Total LPA vs. higher intensities [ilr3]		LPA long vs. short bouts [ilr4]		Total MPA vs. higher intensities [ilr5]		MPA long vs. short bouts [ilr6]		VPA long vs. short bouts [ilr7]		Overall	
	n	β (SE)	*p*	β (SE)	*p*	β (SE)	*P*	β (SE)	*p*	β (SE)	*P*	β (SE)	*p*	β (SE)	*p*	F_(df)_; *p*	Adj R^2^
z-BMI	467	− 0.29 (0.72)	0.69	0.37 (0.52)	0.49	− 0.40 (0.85)	0.64	**1.70** **(0.83)**	0.04	0.54 (0.38)	0.16	**− 1.03** **(0.46)**	0.03	0.04 (0.21)	0.85	4.80_(13,453)_; <0.001	0.10
BF %	400	5.37 (3.27)	0.10	− 2.34 (2.37)	0.32	**− 8.00** **(3.81)**	0.04	**11.20** **(3.70)**	0.003	**4.57** **(1.61)**	0.005	**− 5.28** **(1.90)**	0.006	1.06 (0.90)	0.24	3.04_(13,386)_; <0.001	0.06
z-triceps	427	0.73 (0.80)	0.36	0.43 (0.58)	0.46	− 1.34 (0.94)	0.16	**2.06** **(0.92)**	0.03	**1.15** **(0.42)**	0.007	**− 1.03** **(0.50)**	0.04	0.36 (0.24)	0.14	3.28_(13,413)_; <0.001	0.07
z-subscap	418	1.16 (1.04)	0.26	− 0.006 (0.76)	0.99	− 1.52 (1.23)	0.22	2.14 (1.21)	0.07	0.77 (0.56)	0.16	− 0.97 (0.67)	0.14	0.25 (0.31)	0.42	2.13_(13,404)_; 0.01	0.01
SBP	309	− 7.29 (8.19)	0.37	**12.45** **(6.06)**	0.04	1.18 (9.75)	0.90	− 3.82 (9.93)	0.70	2.77 (4.69)	0.55	− 6.01 (5.66)	0.29	− 0.34 (2.70)	0.90	1.34_(13,295)_; 0.19	0.01
DBP	309	− 6.96 (6.67)	0.30	**10.80** **(4.93)**	0.03	2.49 (7.93)	0.75	− 1.13 (8.09)	0.89	1.94 (3.82)	0.61	− 4.84 (4.61)	0.29	0.16 (2.20)	0.94	1.38_(13,295)_; 0.17	0.02
HR	309	− 1.48 (8.99)	0.87	10.05 (6.66)	0.13	2.02 (10.70)	0.85	− 1.10 (10.91)	0.92	5.01 (5.15)	0.33	− 2.62 (6.22)	0.67	2.34 (2.96)	0.43	2.81_(13,294)_; <0.001	0.07
PWV	340	− 1.00 (0.48)	0.83	0.38 (0.35)	0.27	− 0.01 (0.57)	0.99	− 0.30 (0.57)	0.59	0.13 (0.26)	0.62	− 0.15 (0.32)	0.64	0.04 (0.15)	0.77	1.09_(13,326)_; 0.36	0.004
aIMT	310	− 0.004 (0.006)	0.47	0.002 (0.004)	0.66	0.002 (0.007)	0.79	0.0001 (0.003)	0.99	0.001 (0.003)	0.66	0.002 (0.004)	0.68	0.002 (0.002)	0.21	1.92_(13,296)_; 0.03	0.04
cIMT	373	0.001 (0.005)	0.76	0.002 (0.004)	0.54	− 0.002 (0.006)	0.66	0.003 (0.006)	0.66	0.0002 (0.003)	0.93	0.001 (0.003)	0.72	0.001 (0.002)	0.40	1.94_(13,359)_; 0.02	0.03

Models adjusted for sex, age, maternal education, total energy intake, and birth weight z-score. The [*ilr*] coordinates 1, 3 and 5 correspond to relative proportions of SED, LPA, and MPA versus the remaining (higher/more intense) intensities, respectively; [*ilr*] coordinates 2, 4, 6 and 7 represent isometric log-ratios of simplex coefficients of long (> 1 min) versus short bouts within one intensity, keeping the total time within the respective intensity constant. Bold font indicates statistical significance

Adj, adjusted; aIMT, mean far aortic intima-media thickness; BF, body fat; cIMT, maximum far carotid intima-media thickness; DBP, diastolic blood pressure; HR, heart rate; *ilr*, isometric log-ratio; LPA, light-intensity physical activity; MPA, moderate-intensity physical activity; PWV, pulse wave velocity; SBP, systolic blood pressure; SE, standard error; SED, sedentary time; VPA, vigorous-intensity physical activity; z-BMI, body mass index z-score; z-subscap, subscapular skinfold z-score; z-triceps, triceps skinfold z-score

The ‘Goldilocks Day’ models indicated that optimal daily waking durations for the lowest 5% of percent body fat (excluding participants who were ‘too thin’) were: 6 h of sedentary time (76% spent in long bouts); 4 h in LPA (41% in long bouts), 60 min in MPA (10% in long bouts), and 32 min in VPA (3% in long bouts). To compare, the predictions for the highest 5% of percent body fat were: 5 h of sedentary time (74% spent in long bouts), 4.7 h of LPA (55% in long bouts), 60 min in MPA (11% in long bouts), and 14 min in VPA (14% in long bouts).

## Discussion

This study aimed to examine the association of the daily time-use compositions of SED and PA patterns with cardiometabolic risk factors in 4-year-old children. Participants spent on average almost half of their waking time in SED, with most of that time spent in long bouts. By comparison, little time was spent in higher intensities of PA, with 10% of the day spent in total volume of MPA and < 4% in total volume of VPA (of which most was accumulated in shorter bouts). A higher ratio of long vs. short LPA bouts were associated with increased adiposity, whereas a higher ratio of long vs. short MPA bouts were associated with decreased adiposity. Additionally, a higher ratio of total volume of MPA versus VPA was associated with increased adiposity.

The overall waking time-use composition was comparable with two previous studies, although children in the current study spent slightly less time sedentary and slightly more time being physically active. Carson et al. [12] reported that 3- to 4-year-old children spent the equivalent of 60%, 31% and 9% of waking time in SED, LPA and MVPA, respectively (when sleep time was removed). Taylor et al. [13] reported that 5-year-old children spent 60%, 36% and 4% of their waking time in SED, LPA and MVPA, respectively. Both of those studies used a 24-h wear protocol; it is reassuring that our results are consistent despite the different wear protocols. In addition, our study provides more insight into the composition of children’s movement behaviors with the inclusion of patterns.

Our findings showed that a higher ratio of long versus short LPA bouts was associated with higher adiposity markers (i.e., higher zBMI, percent body fat and triceps skinfold z-score). Verswijveren et al. [15] also found that higher levels of continuous LPA relative to sporadic LPA (i.e., long versus shorter bouts) were associated with higher estimated zBMI and waist circumference in school-aged children. This could potentially be attributed to the possible benefits of shorter bouts of LPA breaking up SED; evidence shows that changes in posture (i.e. moving from a sitting to standing position) are associated with increased energy expenditure in preschool aged children [35]. However, no associations were observed for short versus long bouts of SED in the current study. Alternatively, it may be that short bouts of LPA are dips below the MVPA threshold, or that long bouts of LPA are the sole activity for some children, who are then not engaging in sufficient higher intensity activity to obtain the health benefits. Our ‘Goldilocks Day’ findings showed that children in the highest and lowest 5% of percent body fat measurements spent 55% and 41% of their LPA in long bouts, respectively, further highlighting that long bouts of LPA may be the sole activity for some children.

Conversely, a higher ratio of long vs. short MPA bouts was inversely associated with adiposity markers (i.e., lower zBMI, percent body fat and triceps skinfold z-score), suggesting that encouraging young children to engage in MPA for longer periods of time (rather than their usual sporadic PA) may be important for health. Additionally, our finding that the total volume of MPA vs. VPA was positively associated with adiposity markers (i.e., higher percent body fat and triceps skinfold z-score) suggests that higher intensity PA should be encouraged. Findings from our ‘Goldilocks Day’ model associations add further weight to this argument. These findings suggest that even though SED was lower, LPA was higher, and MPA nearly identical in children in the highest 5% of percent body fat measurements (compared to those in the lowest 5%), the main impact on this outcome seemed to come from VPA, which was nearly twice as much in children with optimal body fat percentages compared to those with the highest scores. This underscores the importance of the inclusion of one hour of ‘energetic play’ (operationalized as MVPA) in the 24-hour movement guidelines for preschool aged children [34]. However, our ‘Goldilocks Day’ included >100 min of MVPA combined, which is similar to Dumuid et al.’s findings that the overall Goldilocks Day for 11- to 12-year-old included 1.5 h of MVPA [33]. Recommendations for PA may need to be considered in light of these findings, with higher amounts of MVPA recommended for children; however, intervention trials are needed to determine dose-response.

Outside of the associations observed for measures of adiposity, we found no associations between individual *ilr* coordinates (i.e., patterns) and other cardiometabolic risk factors including pulse wave velocity, systolic blood pressure, diastolic blood pressure, heart rate, aortic intima-media thickness and carotid intima-media thickness. Very few existing studies have examined cardiometabolic risk factors beyond adiposity in this population [36]. Of those that have, one study reported that total volume of PA appeared to have an indirect association with blood lipids and lipoproteins in children aged 3–4 years [37]. This indirect association appeared to be via lower levels of body fatness and higher levels of fitness, but no direct association was observed [37]. Another study in slightly older children (5–7 years) reported an inverse association between total volume of PA and diastolic blood pressure [38]. No studies have examined the impact of patterns of PA and SED on these cardiometabolic risk factors. A review of activity patterns and cardiometabolic risk factors in children and adolescents found some evidence of associations, although overall conclusions could not be made given substantial variety in pattern definitions and the types of outcomes measured [3]. Given that we observed few associations in preschoolers, it may be that their short lifetime exposure to PA and SED patterns means that there has not been any impact yet on the more “advanced” measures of cardiometabolic risk.

Strengths of our study include the use of accelerometers to measure SED and PA and the inclusion of a large range of cardiometabolic risk factors, measured using standardized protocols, not previously investigated in children of this age. Additionally, our study utilized novel statistical methods, incorporating patterns of SED and PA into compositional analyses, which have previously only been used once in older children and adolescents [15]. Despite these strengths, our study has several limitations. First, accelerometers were only worn during waking hours and, as such, we could not include sleep in analyses. Given there are potentially important differences between sleep and non-wear time, and because we know sleep is important for young children’s health [39] and is an important component of the 24-hour day, future studies should consider using 24-h accelerometer wear protocols. Second, there is limited validity of waist-worn accelerometers for measuring SED bout durations [40], especially in young children. As such, future studies should consider the use of posture-based devices to assess young children’s SED or sitting time. Third, the data were cross-sectional, so we are unable to infer causality. In addition, the estimated differences in cardiometabolic risk factors cannot be directly interpreted as an effect of time reallocation from one component (of the time-use composition) to another. Longitudinal studies are needed to investigate the long-term health effects of reallocation of activity patterns. Fourth, the sample comprised a high percentage of highly educated mothers, recruited from one region in Victoria, Australia, so the generalizability of our findings may be limited. Finally, given the exploratory nature of our study, many tests were performed without significance adjustment; therefore, there is an increased likelihood of false discovery due to multiple testing.

## Conclusion

This study is among the first to examine associations between patterns of physical activity and sedentary behavior and a range of cardiometabolic risk factors in preschool-aged children using compositional data analysis. Our findings suggest that how movement behaviors are accumulated – such as the duration and intensity of activity bouts—may be important for cardiometabolic health, beyond total volume alone. From a practical perspective, these results highlight the potential value of encouraging preschoolers to engage in more vigorous-intensity physical activity throughout the day, and to sustain moderate-intensity activity for at least a minute at a time when possible. Early childhood educators, health professionals, and parents may consider incorporating short, high-intensity play opportunities into daily routines to support healthier cardiometabolic profiles in young children.

## Supplementary Information

Below is the link to the electronic supplementary material.

Supplementary Material 1

Supplementary Material 2

## Data Availability

Barwon Infant Study (BIS) data requests are considered on scientific and ethical grounds by the BIS Steering Committee. If approved, data are provided under collaborative research agreements.

## Abbreviations

aIMT: Aortic intima–media thickness
BMI: Body mass index
cIMT: Carotid intima–media thickness
ilr: Isometric log ratio
LPA: Light–intensity physical activity
MPA: Moderate–intensity physical activity
MVPA: Moderate–to vigorous–intensity physical activity
SED: Sedentary time
VPA: Vigorous–intensity physical activity
