# Supplementary material for: Physical activity and sedentary pattern compositions and cardiometabolic risk in preschoolers
Source: J Act Sedentary Sleep Behav. 2025 Dec 1;4:20. doi: 10.1186/s44167-025-00090-y (PMC12667165; doi:10.1186/s44167-025-00090-y)
Supplement: Supplementary file 2 — Supplementary Material 2 [file 44167_2025_90_MOESM2_ESM.docx]

Supplementary Table 1. Sequential binary partition sign matrix for time-use compositions expressed as isometric log-ratio (*ilr*) coordinates^a^

| *ilr* coordinate | x_1_  SED (Long) | x_2_  SED (Short) | x_3_  LPA (Long) | x_4_  LPA (Short) | x_5_  MPA (Long) | x_6_  MPA (Short) | x_7_  VPA (Long) | x_8_  VPA (Short) | **(Variable name) Interpretation^b^** |
| --- | --- | --- | --- | --- | --- | --- | --- | --- | --- |
| z_1_ | +1 | +1 | -1 | -1 | -1 | -1 | -1 | -1 | **(*ilr* 1)** Total SED vs. total LPA, total MPA, and total VPA |
| z_2_ | +1 | -1 | 0 | 0 | 0 | 0 | 0 | 0 | **(*ilr* 2)** Long SED vs. short SED |
| z_3_ | 0 | 0 | +1 | +1 | -1 | -1 | -1 | -1 | **(*ilr* 3)** Total LPA vs. total MPA and total VPA |
| z_4_ | 0 | 0 | +1 | -1 | 0 | 0 | 0 | 0 | **(*ilr* 4)** Long LPA vs. short LPA |
| z_5_ | 0 | 0 | 0 | 0 | +1 | +1 | -1 | -1 | **(*ilr* 5)** Total MPA vs. total VPA |
| z_6_ | 0 | 0 | 0 | 0 | +1 | -1 | 0 | 0 | **(*ilr* 6)** Long MPA vs. short MPA |
| z_7_ | 0 | 0 | 0 | 0 | 0 | 0 | +1 | -1 | **(*ilr* 7)** Long VPA vs. short VPA |

Notes: Long bouts were defined as >1-min bouts and short bouts as the time accumulated in ≤1-min bouts

^a^ Basis selected to evaluate bout duration (long *vs.* short) for each activity intensity.

^b^ All *ilr* coordinates are on the natural log scale and evaluate either time uses or geometric means for each individual of multiple time uses. For example, the first *ilr* coordinate refers to the geometric mean of time in long and short bouts of SED relative to the geometric mean of long and short LPA, MPA, and VPA, *etc*.

Abbreviations: *ilr =* isometric log-ratio; LPA = light-intensity physical activity; MPA = moderate-intensity physical activity; SED = sedentary time; VPA = vigorous-intensity physical activity
